# Effect of facet joint distraction on the functional and radiological outcomes after anterior cervical disc replacement

**DOI:** 10.1186/s12891-022-05705-y

**Published:** 2022-08-03

**Authors:** Chunyi Yan, Hong Wang, Tingkui Wu, Chengyi Huang, Haimiti Abuduaini, Beiyu Wang, Hao Liu

**Affiliations:** Department of Orthopedics, West China Hospital, Sichuan University, #37 Guoxue Alley, Wuhou District, Chengdu, Sichuan Province People’s Republic of China

**Keywords:** Cervical spondylotic radiculopathy, Cervical disc replacement, Facet joint distraction, Functional outcome, Radiological outcome

## Abstract

**Objective:**

The purpose of this study is to explore: 1) whether the extent of facet joint distraction affects functional outcomes following single-level anterior cervical disc replacement (ACDR) for cervical spondylotic radiculopathy and 2) whether the extent of facet joint distraction correlates with the cervical sagittal parameters.

**Methods:**

We performed a retrospective analysis on 70 patients who had undergone a single-level ACDR to treat cervical spondylotic radiculopathy between January 2014 and December 2018. Pre- and post-operative lateral cervical spine X-ray radiographs were collected to determine radiographic parameters, including C0-C2 angle, C2-C7 angle, C7 Slope (C7S), T1 Slope (T1S), C2–C7 sagittal vertical axis (SVA), C2-C7 range of motion (ROM), Segmental ROM, disc height (DH) and inter-facet distance (ID). And the extend of facet joint distraction was evaluated by the two indexes: degree of intervertebral distraction (DID) defined and degree of facet joint distraction (DFJD). The visual analog scale (VAS_neck_) and the Neck Disability Index scores (NDI) were adopted to demonstrate functional outcomes. Patients with the functional outcome improvement below the average were set as the positive group in the receiver operating characteristic (ROC) curve analysis, to find an optimal cut-off value of extent of facet joint distraction.

**Results:**

VAS_neck_ and NDI scores improved significantly from pre- to post-operation among the entire cohort, and DFJD had a statistically significant negative correlation with ΔVAS_neck_ (*p* < 0.001) and ΔNDI (*p* < 0.001). According to ROC curve analysis, the cut-off value of DFJD for differing the appropriate and excessive distraction groups was set at 29.16% (sensitivity = 70.73%, specificity = 67.86%). Between these two groups, the ΔT1S, ΔROM, ΔVAS_neck_, and ΔNDI were significantly different (*p* < 0.05).

**Conclusion:**

Single-segment ACDR may improve the functional outcome of patients with cervical spondylotic radiculopathy. However, those whose DFJD was greater than 29.16% had worse VAS_neck_ and NDI scores, as well as a lower ΔT1S and a lower ΔROM.

## Background

In an aging society, the number of people affected by cervical spondylosis is gradually increasing. Patients with cervical spondylosis typically experience numbness and radiating discomfort in their neck and upper limbs as a result of the compression caused by a degenerated intervertebral disc. These manifestations may have a minor impact on the patient's quality of life and ability to work. Surgery is another viable option if conservative therapy fails or the compression worsens. At present anterior approaches such as anterior cervical disc replacement (ACDR) and anterior cervical discectomy and fusion (ACDF) have demonstrated great effectiveness. Following cervical surgery, the facet joint may be damaged, resulting in postoperative neck pain [1]. As a non-fusion technique, ACDR is effective at decompressing the nerve root and spinal cord while preserving the physiological functions of the cervical spine, as evidenced by an acceptable disparity in facet joint contact pressure before and after the operation [2].

According to a study by Lin [3], in ACDF, the distraction of the facet joint had a negative correlation with functional outcome, with a poor functional outcome indicated by an inter-facet distance greater than a specific degree. The sagittal sequence of the cervical spine is critical for its fundamental functions [3]. Misalignment of the cervical vertebrae can impair spinal functioning and even have a significant detrimental influence on the quality of life [4]. As critical factors for determining the cervical sagittal balance [5, 6], cervical sagittal plane parameters are widely employed in evaluating cervical surgery, while some of them show a correlation with the postoperative functional score [3]. Considering that the mobility of facet joints will be preserved after non-fusion surgery, it is more important to explore the changes of facet joints after ACDR. However, no articles currently report the association between facet joint distraction and clinical outcomes in ACDR.

Therefore, the purpose of this study is to determine: (1) whether the extent of facet joint distraction has an effect on the functional outcome of single-level ACDR for cervical spondylotic radiculopathy and (2) whether the extent of facet joint distraction correlates with cervical sagittal parameters.

## Materials and methods

### Study design

A retrospective study of patients who have undergone single-level ACDR with the Prestige-LP implant (Medtronic Sofamor Danek, Memphis, TN) performed by the same orthopedic surgeon to treat cervical spondylotic radiculopathy between January 2014 and December 2018 was performed. All the patients were told about the type and purpose of the study and signed informed consent before participation. This study was submitted and approved by the ethics committee of West China Hospital.

### Study participants

The hospital information system was used to retrieve retrospectively, and there was a total of 235 patients affected by the forementioned condition. Our inclusion criteria were as follows: (1) patients with cervical spondylosis treated with single-level ACDR; (2) patients with a minimum of 12 months follow-up after surgery; and (3) patients with clear lateral cervical radiographs that could be seen and accurately measured, particularly for the facet joints. Patients were excluded from this study if they: (1) had any other neurological symptoms or extraspinal cervical lesions such as thoracic outlet syndrome, humeral epicondylitis, carpal tunnel syndrome, or cubital tunnel syndrome; or (2) with severe facet joint disease or degeneration; or (3) with incomplete imaging or follow-up data.

Finally, a total of 70 patients were eligible to participate in the study, and we recorded data on their gender, age, height, weight, body mass index (BMI), characteristics of operation, and preoperative and postoperative radiographs.

### Assessment of radiographic outcome

**Fig. 1:**
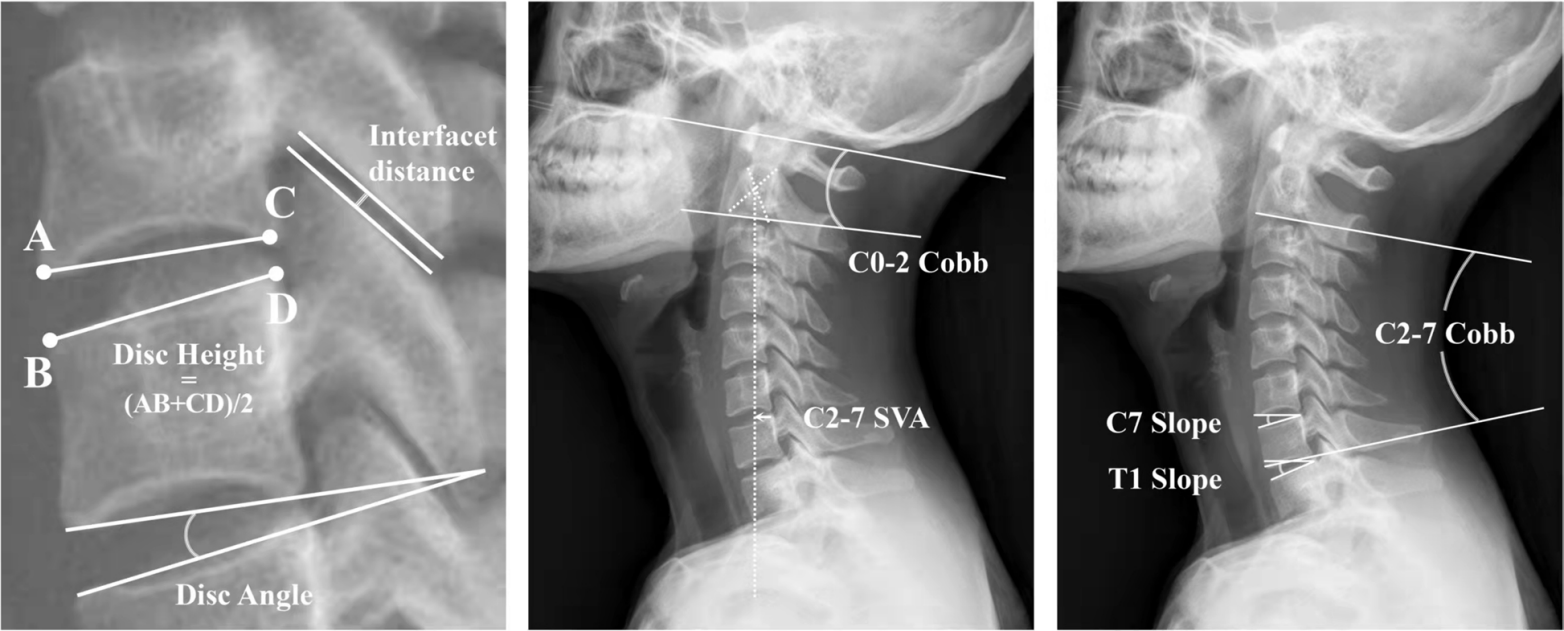
The measuring methods of imaging parameters

To evaluate facet joint distraction, disc height and inter-facet distance were determined through lateral cervical spine X-ray. Certain cervical sagittal parameters that we used were also included in the measurement. Each parameter was defined specifically as follows (Fig. 1):

**C0-C2 angle:** The angle between the McGregor line and the lower endplate of the axis, with ( +) for lordosis and (-) for kyphosis.

**C2-C7 angle:** The angle between the lower endplate of the axis and the lower endplate of C7, with ( +) for lordosis and (-) for kyphosis.

**C2-C7 range of motion (ROM):** The sum of the C2-C7 angle measured in the flexion and extension positions.

**Segmental ROM:** The sum of the disc angle measured in the flexion and extension positions, respectively (disc angle: the angle between the lower endplate of the upper vertebra and the upper endplate of the lower vertebra at the operation segment).

**C7 slope (C7S):** The angle between the horizontal line and the upper endplate of C7.

**T1 slope (T1S):** The angle between the horizontal line and the upper endplate of T1.

**C2–C7 sagittal vertical axis (SVA):** The distance between the posterior, superior corner of C7 and the centroid of C2 on the plumbline.

**Disc height (DH):** The average height of the anterior disc, the middle disc, and the posterior disc**.**

**Inter-facet distance (ID):** Inter-facet distance at the level of artificial cervical disc replacement (ACDR).

The extend of facet joints distraction were assessed by two parameters, which were calculated using following formula:

**Degree of intervertebral distraction (DID)** = 100% × (postoperative DH – preoperative DH) / (preoperative DH).

**Degree of facet joint distraction (DFJD)** = 100% × (postoperative ID – preoperative ID) / (preoperative ID).

All imaging evaluations were independently assessed mainly by two senior orthopedists, and the disagreements were finally determined by consensus, by the third orthopedist.

### Assessment of functional outcome

The neck visual analogue scale (VAS_neck_) and the Neck Disability Index (NDI) were used to assess functional outcomes [7]. All patients had these indexes recorded preoperatively and 12 months postoperatively. 1) Pain intensity quantified using a VAS_neck_ score on a 10-point numeric rating scale ranging from 0 (no pain) to 10 (worst pain imaginable); 2) The NDI score ranged from 0 (no disability) to 100 (maximal disability), encompassing domains such as pain intensity, personal care, lifting, reading, headache, and concentration, etc. In this study, the VAS_neck_ score was considered as the primary index of functional outcome.

### Statistical analysis

All the data were statistically analyzed using IBM SPSS statistics software, version 26.0 (IBM Corp., Armonk, New York, USA). *P* < 0.05 was considered to be statistically significant.

The count data were organized as a sum (%), whereas the normal distribution data were presented as mean ± standard deviation (SD). The paired-sample t-test was used to determine the differences in patients' preoperative and postoperative functional scores and radiographic parameters. The ΔValue was defined as the post-operative value minus the pre-operative value, whereas the ΔVAS_neck_ and ΔNDI used absolute values to measure functional improvement. Pearson correlation analysis was used to determine the correlation between patients' characteristic data, functional scores, and imaging parameters. The receiver operating characteristic (ROC) curve was used to determine the critical value of DFJD, dividing all samples into two groups: appropriate distraction (AD) group and excessive distraction (ED) group.

The Pearson χ^2^ test or the Fisher exact test were used to analyze the inter-group discrepancy for count data such as gender and operation segment. The independent-sample t-test was used to determine normally distributed data such as some characteristic data, functional scores, and imaging parameters.

## Results

### Basal data

Based on the inclusion and exclusion criteria, a total of 70 patients with cervical spondylotic radiculopathy were involved in this study, among whom there were 30 males and 40 females, with a mean age of 42.92 ± 7.95 years and an average BMI of 22.59 ± 2.98. For the details of the operation, the distribution of operation segments among patients was 2 of C2/3, 9 of C3/4, 52 of C4/5, and 7 of C5/6. It took an average time of 111.49 ± 22.25 min for the surgery in general, with a mean blood loss of 47.61 ± 27.19 ml. The follow-up time was 13.72 ± 1.21 after the operation on average (Table 1).

**Table 1:** Characteristics of patients and overall outcome parameters

	ACDR Patients (*n* = 70)
Age, years	42.92 ± 7.95
Male, n (%)	30 (42.85%)
Operation segment, n	
C2/3: C3/4: C4/5: C5/6	2:9:52:7
BMI, kg/m^2^	22.59 ± 2.98
Operation time, mins	111.49 ± 22.25
Blood loss, ml	47.61 ± 27.19
Follow-up time, months	13.72 ± 1.21
C0-C2 angle, º	
Preoperative	20.52 ± 8.73
Postoperative	19.08 ± 7.43
C2-C7 angle, º	
Preoperative	11.50 ± 10.10
Postoperative	15.03 ± 8.68*
C7S, º	
Preoperative	18.47 ± 7.93
Postoperative	20.49 ± 7.45*
T1S, º	
Preoperative	22.56 ± 7.48
Postoperative	23.96 ± 7.49
C2-C7 SVA, mm	
Preoperative	15.82 ± 9.03
Postoperative	16.22 ± 7.49
C2-C7 ROM, º	
Preoperative	50.69 ± 15.54
Postoperative	56.55 ± 14.16*
Segmental ROM, º	
Preoperative	8.44 ± 4.37
Postoperative	9.64 ± 4.75
VAS	
Preoperative	6.10 ± 1.35
Postoperative	1.61 ± 1.03*
NDI	
Preoperative	27.35 ± 4.21
Postoperative	7.04 ± 3.85*
DID, %	45.38 ± 27.77
DFJD, %	30.50 ± 17.95

*BMI* Body mass index, *C7S* C7 slope, *T1S* T1 slope, *SVA* Sagittal vertical axis, *ROM* Range of motion, *VAS* Visual analog scale, *NDI* Neck Disability Index, *DID* Degree of Intervertebral Distraction, *DFJD* Degree of Facet Joint Distraction

Values are expressed as means ± SD

^*^p < 0.05, Significantly different between the preoperative and the postoperative

### Radiographic outcomes

The results of cervical sagittal parameters were as follows: Significant differences between preoperative and postoperative C2-C7 angle (11.50 ± 10.10° vs 15.03 ± 8.68°, *p* = 0.004), between preoperative and postoperative C7 slope (18.47 ± 7.93° vs 20.49 ± 7.45°, *p* = 0.005), between preoperative and postoperative C2-C7 ROM (50.69 ± 15.54° vs 56.55 ± 14.16°, *p* = 0.015) were observed. While there were no significant differences between pre-and post-operation C0-C2 angle, T1 slope, C2-C7 SVA, and Segmental ROM. This demonstrates that single-level ACDR can significantly influence several cervical sagittal parameters, including C2-C7 Cobb, C7 slope, and ROM (Table 1).

The results of the parameters reflecting facet joint distraction were as follows: The average degree of intervertebral distraction (DID) was 45.38 ± 27.77%, and the average degree of facet joint distraction (DFJD) was 30.50 ± 17.95%, indicating that ACDR would influence the facet joint to some extent.

### Functional outcome

The average pre- and postoperative VAS_neck_ scores were 6.10 ± 1.35 and 1.61 ± 1.03, respectively. The average preoperative and postoperative NDI were 27.35 ± 4.21 and 7.04 ± 3.85, respectively.

Both VAS_neck_ (*p* < 0.001) and NDI (*p* < 0.001) scores significantly improved in patients who underwent ACDR. Additionally, ACDR had a positive effect on relieving pain and restoring function (Table 1).

### Pearson correlation analysis

Pearson correlation analysis was used to determine the correlation between patients' characteristic data, changes in radiographic outcome, and functional outcomes as a result of the ACDR.

It was shown that DFJD was significantly correlated with BMI (*p* < 0.05). And for the functional outcome, there was a significant correlation between ΔVAS_neck_ and ΔNDI (*p* < 0.01). Additionally, ΔVAS_neck_ and ΔNDI were statistically significant in relation to the DFJD (*p* < 0.01 for either), but not the DID. Besides, the ΔC2-C7 angle was significantly correlated with both the ΔC7S (*p* < 0.01) and ΔC2-C7 ROM (*p* < 0.05) within the sagittal parameters, there was also a significant correlation between ΔC7S and ΔC2-C7 ROM (*p* < 0.01) (Table 2).

**Table 2:** Pearson correlation coefficients and *p* values

	Age	BMI	Operation Time	ΔC2-C7 angle	ΔC7S	ΔC2-C7 ROM	ΔVAS	ΔNDI	DID	DFJD
Age	×	0.225	0.006	0.068	-0.029	-0.148	-0.171	0.091	-0.036	0.073
BMI		×	-0.069	-0.178	-0.223	-0.143	0.091	0.070	0.003	**-0.241**^a^
Operation Time			×	0.120	-0.084	-0.224	-0.205	-0.187	0.120	0.190
ΔC2-C7 angle				×	**0.487**^b^	**0.298**^a^	0.218	0.100	**0.278**^a^	-0.016
ΔC7S					×	**0.390**^b^	0.205	0.148	0.098	-0.075
ΔC2-C7 ROM						×	0.214	0.076	0.046	-0.030
ΔVAS							×	**0.308**^b^	0.017	**-0.534**^b^
ΔNDI								×	0.120	**-0.380**^b^
DID									×	0.224
DFJD										×

BMI, body mass index; C7S, C7 slope; ROM, range of motion; VAS, visual analog scale; NDI, neck disability index; DID, degree of Intervertebral Distraction; DFJD, degree of Facet Joint Distraction

^a^Correlation is significant at the 0.05 level (2-tailed)

^b^Correlation is significant at the 0.01 level (2-tailed)

### ROC curve analysis

**Fig. 2:**
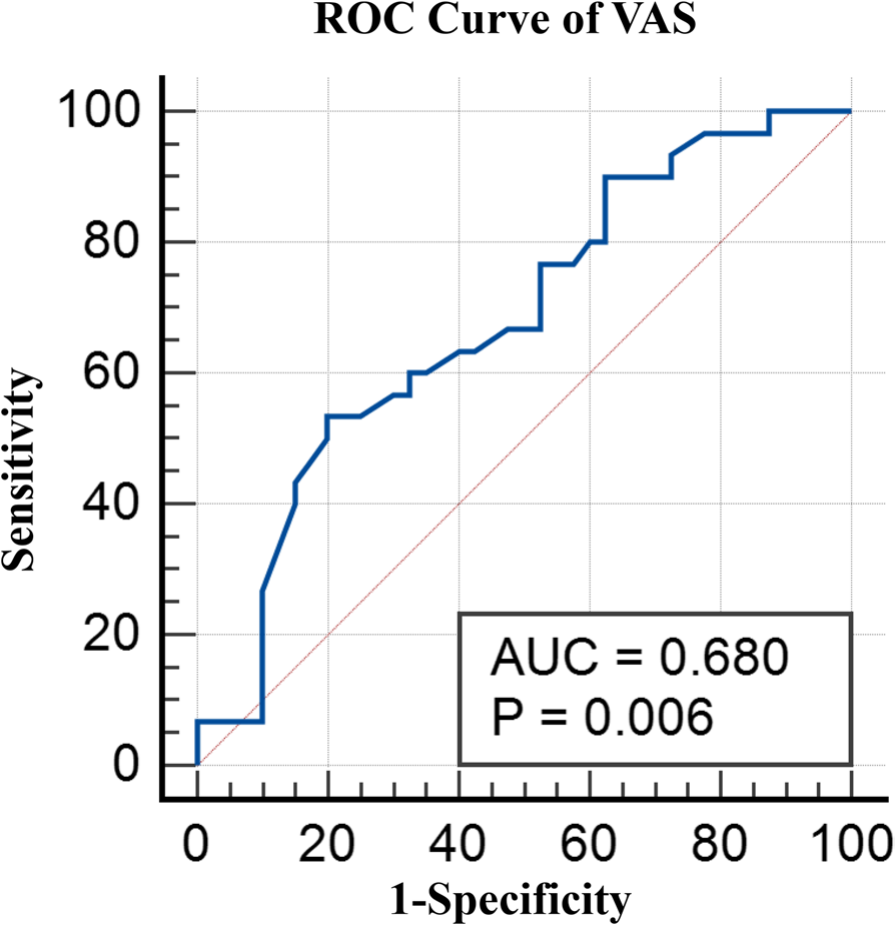
Receiver operating characteristic curve of VAS

**Fig. 3:**
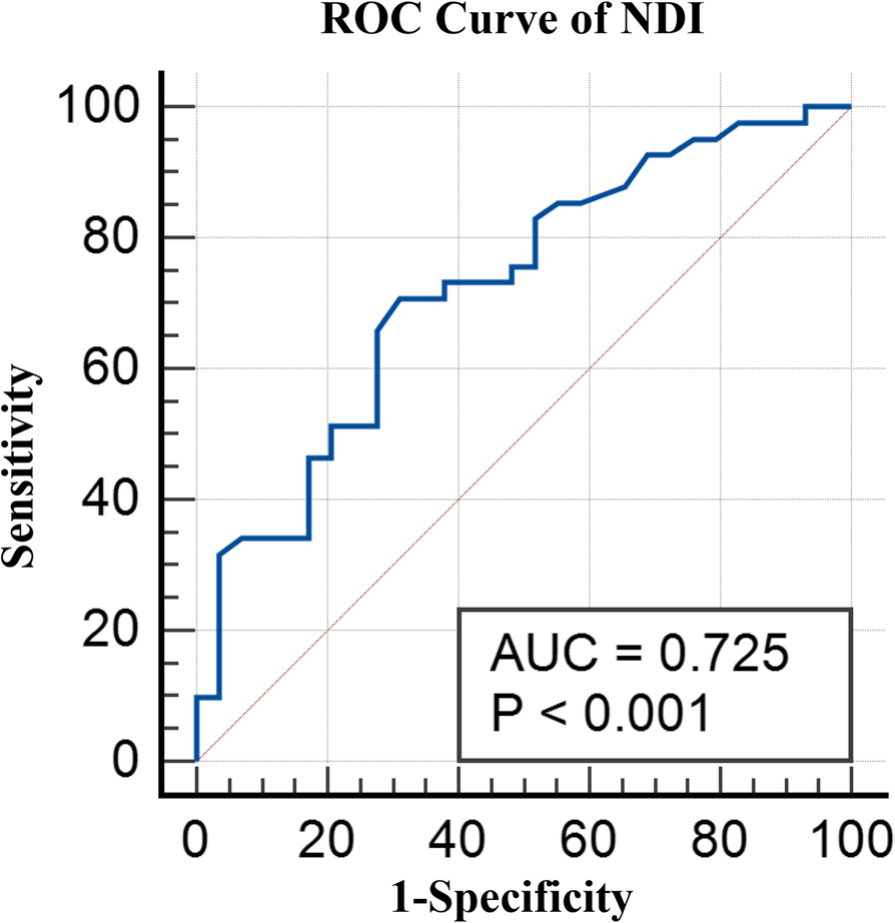
Receiver operating characteristic curve of NDI

The ROC Curve Analysis was used to determine the correlation between the DFJD with the ΔVAS_neck_ and ΔNDI, respectively. Patients whose ΔVAS_neck_ or ΔNDI was less than the mean value for all patients were classified as positive groups, indicating that their functional outcome improvement was less than the mean condition of the cohort. For the ΔVAS_neck_, the area under the curve (AUC) was 0.68 (SE 0.06, 95% confidence interval [CI] 0.55–0.78, *p* = 0.006) (Fig. 2). For the ΔNDI, the AUC was 0.72 (SE 0.06, 95% CI 0.60–0.82, *p* < 0.001) (Fig. 3). Thus, the ΔNDI was taken as the primary functional outcome in the following analysis, and the ROC curve of ΔNDI indicated that the cut-off value of the DFJD was 29.16%, which had a sensitivity of 70.73%, specificity of 68.97%.

The patients were divided into two groups: AD and ED groups, based on the cut-off value of DFJD determined by the ROC analysis. The results revealed that there were significant differences in ΔT1S, ΔC2-C7 ROM, ΔVAS_neck,_ and ΔNDI between the two groups, but no significant differences in age, gender, operation segment, BMI, ΔC0-C2 angle, ΔC2-C7 angle, ΔC7S, ΔC2-C7 SVA and ΔSegmental ROM between the two groups (Table 3).

**Table 3:** Comparison of patient profile, imaging parameters, and outcome scores grouped by DFJD

	AD Group	ED Group	*P*-value
No. of patients	38	32	
Age, years	42.10 ± 7.74	43.90 ± 8.20	0.064
Male, n (%)	16 (47.05%)	14 (38.88%)	0.349
Operation segment, n			
C2/3: C3/4: C4/5: C5/6	2:3:25:4	0:6:27:3	0.367
BMI, kg/m^2^	23.02 ± 3.25	22.77 ± 2.59	0.189
ΔC0-C2 angle, °	-1.87 ± 7.45	-0.92 ± 8.17	0.612
ΔC2-C7 angle, °	4.94 ± 10.97	1.83 ± 8.13	0.190
ΔC7S, °	3.03 ± 6.43	0.81 ± 4.99	0.118
ΔT1S, °	3.30 ± 5.66	-0.99 ± 7.10	0.005*
ΔC2-C7 SVA, mm	-0.06 ± 0.86	0.16 ± 0.63	0.225
ΔC2-C7 ROM, °	10.33 ± 21.00	0.52 ± 16.87	0.037*
ΔSegmental ROM, °	2.16 ± 2.05	0.06 ± 5.05	0.089
ΔVAS	5.13 ± 1.16	3.81 ± 1.46	0.000*
ΔNDI	21.68 ± 2.76	18.87 ± 3.67	0.001*

*AD* Appropriate distraction, *ED* Excessive distraction, *BMI* Body mass index, *VAS* Visual analog scale, *NDI* Neck Disability Index, *DH* Disc height, *C7S* C7 slope, *T1S* T1 slope, *SVA* Sagittal vertical axis

Values are expressed as means ± SD

^*^p < 0.05, Significantly different

## Discussion

ACDR has a similar effect to ACDF in the treatment of cervical spondylotic radiculopathy and may even be superior in some clinical outcomes [8, 9], particularly in terms of cervical mobility and avoidance of adjacent segment disease [10]. Both of them use a similar surgical technique that uses the anterior cervical approach to remove the degenerated intervertebral disc and implant the prosthetic to maintain the intervertebral height and achieve decompression of nerve [11]. However, both surgeries can potentially expand the intervertebral space and alter the facet joint distance. In the ACDF, Lin et al. [3] investigated the relationship between inter-facet distance and VAS scores, cervical sagittal parameters, in 68 patients who had undergone ACDF for cervical spondylotic radiculopathy. They hypothesized that patients had less improvement in VAS if their inter-facet distance group had changes greater than 0.7 mm (ΔVAS of 2.60 ± 1.27 for the appropriate distraction group and ΔVAS of 1.55 ± 2.42 for the excessive distraction group). On the other hand, the data demonstrated that increasing the inter-facet distance did not affect cervical sagittal characteristics. As a result, Kirzner et al. [12] enrolled 155 patients who had undergone ACDF to determine if the facet joint distraction altered VAS and NDI values. The final result demonstrated a correlation between changes in inter-facet distance and both VAS (Spearman correlation coefficient 0.52; *p* < 0.001) and NDI (Spearman correlation coefficient 0.34; *p* < 0.001). There was an obvious transition point between the index scores at the 0.3 mm increment in facet joint distance, at which a worse VAS and NDI result would be observed. The research mentioned above confirmed that excessive stress on the facet joints results in a poor prognosis following anterior cervical surgery.

However, no current study has been published discussing whether facet joint distraction affects functional outcomes following ACDR. As far as we know, this is the first study to investigate this correlation in ACDR.

To rule out the influence of multisegmental, multi-operative methods and prosthesis differences on parameter measurement, our study included only patients who underwent single-level ACDR and received Prestige-LP as a prosthesis for the treatment of cervical spondylotic radiculopathy. Considering the changes between preoperative and postoperative intervertebral and facet joint distance were small. We adopted the relative values, DID and DFJD to describe their changes more accurately.

Pearson correlation analysis demonstrated a significant effect of facet joint distraction, quantified by DFJD on VAS_neck_ scores and the NDI index. And an increased distraction results in less improvement in VAS_neck_ and NDI. However, As the other of the two indexes for evaluating facet joint distraction, DID showed no significant correlation with either functional outcome.

Then the patients were divided into AD and ED groups, using a critical DFJD of 29.16% determined by the ROC analysis. Inter-group comparisons revealed a significant difference in average ΔVAS_neck_ (*p* < 0.001) and ΔNDI (*p* = 0.001) between the two groups, indicating that patients whose DFJD was more than 29.16% would have less improvement in their VAS_neck_ and NDI scores. As for the radiological parameter, the AD group had a significantly greater T1 slope increment than the ED group. It was suggested that a higher T1 slope contributes to increased cervical stability [5] and an appropriate distraction distance may result in better neck stability. The ROM increment of AD group was significantly greater than that of ED group, which may attribute to the better improvement of NDI in this group.

The present research on the change of facet joint under ACDR had two opposing views. Bauman et al. [2] measured the C5-C6 facet joint in seven osteoligamentous cadaveric cervical spines before and after implantation of a ProDisc-C at that level. Despite a significant improvement in ROM, they found no significant difference in the facet joint. On the contrary, Wang et al. [13] suggested that the prosthesis may cause a distraction of the facet joint, leading to a change in facet contact pressure. Our study verified Wang's finding that the facet joint was more or less distracted during ACDR, which may result in clinical outcome differentiation. Furthermore, numerous basic investigations demonstrate an association between facet joints and the occurrence of neck pain, with the intervertebral facet joint being regarded as a significant cause of neck pain in addition to nerve compression by osteophytes or the release of inflammatory mediators [14]. The anatomy investigations established that both humans and rats had pain nerve fibers in the minor joint [15]. Additionally, a neurobiology study verified the presence of significant sensory and autonomic nerve fibers innervation of the facet joint capsule, which serves as the structural basis for pain perception [16]. Additional studies confirmed the high sensitivity of the receptors in the facet joint to mechanical stimulation [17]. Additionally, animal investigations demonstrated that there is a threshold load for tensile forces applied to the C6/C7 facet joint and that loads greater than this threshold may cause persistent pain [18]. Therefore, it has been hypothesized that changes in the mechanism and morphology of the facet joint, such as over distraction or the insertion of enlarged implant, can result in postoperative neck pain or even disability [12].

Based on the foregoing, we concluded that in the clinical setting, the Δinter-facet distance might be used as a predictor of functional results, and the diagnostic performance occurs when the ΔDFJD exceeds 29.16%. On the other hand, the results highlighted the detrimental effect of facet joint over distraction, implying the importance of selecting an appropriate prosthesis size [19] and maintaining an appropriate distraction of the facet joint during ACDR to avoid postoperative neck pain and improve T1 slope while maintaining adequate cervical stability.

This study has certain limitations that need to be addressed in this work. First, this is not a prospective randomized controlled study but a retrospective one, and patients who did not undergo surgery but received conservative treatment were excluded. Additionally, precise inter-facet distance measurement needs high-quality X-ray images, which limited the study to only 70 cases and a 1-year follow-up. The relevant radiographic parameters, such as the T1 slope, necessitate a long-term follow-up. What's more, different designs of artificial discs have different effects on facet joints. Prestige-LP, a semiconstrained designed artificial disc, was used as implants in the entire cohort of this study, which may lead to differences with other prostheses. Finally, we hypothesized a correlation between DH change and facet joint distraction, such that a larger prosthesis would result in a significant increase in inter-facet distance. However, the results indicated a negative result.

## Conclusion

In this study, single-segment ACDR may improve the functional outcome of patients with cervical spondylotic radiculopathy. However, those whose degree of facet joint distraction was greater than 29.16% had worse VAS_neck_ and NDI scores. T1S increased in the appropriate distraction group in terms of cervical sagittal parameters. These findings suggest that facet joint distraction should be considered during the surgery.

## Data Availability

The datasets used and analyzed during the current study are available from the corresponding author on reasonable request.

## Abbreviations

ACDR: Anterior cervical disc replacement
ACDF: Anterior cervical discectomy and fusion
C7S: C7 Slope
T1S: T1 Slope
SVA: Sagittal vertical axis
ROM: Range of motion
DH: Disc height
ID: Inter-facet distance
DID: Degree of intervertebral distraction
DFJD: Degree of facet joint distraction
VAS: Visual analog scale
NDI: Neck Disability Index scores
ROC: Receiver operating characteristic
AUC: Area under the curve
SD: Standard deviation
AD: Appropriate distraction
ED: Excessive distraction
